# Assessing Banks' Distress Using News and Regular Financial Data

**DOI:** 10.3389/frai.2022.871863

**Published:** 2022-06-02

**Authors:** Paola Cerchiello, Giancarlo Nicola, Samuel Rönnqvist, Peter Sarlin

**Affiliations:** ^1^Department of Economics and Management, University of Pavia, Pavia, Italy; ^2^Turku Centre for Computer Science, Data Mining Lab, TurkuNLP, University of Turku, Turku, Finland; ^3^Hanken School of Economics, Helsinki, Finland

**Keywords:** credit risk, text analysis, deep learning, Doc2Vec, classification model

## Abstract

In this paper, we focus our attention on leveraging the information contained in financial news to enhance the performance of a bank distress classifier. The news information should be analyzed and inserted into the predictive model in the most efficient way and this task deals with the issues related to Natural Language interpretation and to the analysis of news media. Among the different models proposed for such purpose, we investigate a deep learning approach. The methodology is based on a distributed representation of textual data obtained from a model (Doc2Vec) that maps the documents and the words contained within a text onto a reduced latent semantic space. Afterwards, a second supervised feed forward fully connected neural network is trained combining news data distributed representations with standard financial figures in input. The goal of the model is to classify the corresponding banks in distressed or tranquil state. The final aim is to comprehend both the improvement of the predictive performance of the classifier and to assess the importance of news data in the classification process. This to understand if news data really bring useful information not contained in standard financial variables.

## 1. Introduction

Natural Language Processing (NLP), the interpretation of text by machines, is a complex task due to the richness of human language, its highly unstructured form and the ambiguity present at many levels, including the syntactic and semantic ones. From a computational point of view, processing language means dealing with sequential, highly variable and sparse symbolic data, with surface forms that cover the deeper structures of meaning.

Despite these difficulties, there are several methods available today that allow for the extraction of part of the information content present in texts. Some of these rely on hand crafted features, while others are highly data-driven and exploit statistical regularities in language. Moreover, once the textual information has been extracted, it is possible to enhance it with contextual information related to other sources different from text. The introduction of contextual information in the models is not always a straightforward process but requires a careful choice of the additional information provided in order to not increase noise by using irrelevant features. To accomplish such purpose, there are several methods of variable selection (Guyon and Elisseeff, 2003) that can guide in the choice of the additional features for the model. The recent advancements in text analytic and the addition of contextual information aim at increasing the potential value of text as a source in data analysis with a special emphasis on financial applications (see for example Nyman et al., 2015). In this work, we focus on the issues of understanding and predicting banks distress, a research area where text data hold promising potential due to the frequency and information richness of financial news. Indeed, central banks have already recognized the usefulness of textual data in financial risk analytic (Bholat et al., 2015; Hokkanen et al., 2015; Nymand-Anderson, 2021).

If we focus only on the elicitation of information from textual data, we can find that among the several statistical methods, many rely on word representations. Class based models, for example, learn classes of similar words based on distributional information, like Brown clustering (Brown et al., 1992) and Exchange clustering (Martin et al., 1998; Clark, 2003). Soft clustering methods, like Latent Semantic Analysis (LSA) (Landauer et al., 1998) and Latent Dirichlet Allocation (Blei et al., 2003; Cerchiello et al., 2018), associate words to topics through a distribution over words of how likely each word is in each cluster. In the last years many contributions employ machine learning and semantic vector representations (Mikolov et al., 2013; Pennignton et al., 2014), lately using Long Short-Term Memory (LSTM) networks (Hochreiter and Schmidhuber, 1997; Socher et al., 2013; Cho et al., 2014) to model complex and non-local relationships in the sequential symbolic input. Recursive Neural Tensor Networks (RNTN) for semantic compositionality (Socher et al., 2011, 2013) and also convolutional networks (CNN) for both sentiment analysis (Collobert et al., 2011) and sentence modeling (Kalchbrenner et al., 2014). In this vein, (Mikolov, 2012; Mikolov et al., 2013) and (Pennignton et al., 2014) have introduced unsupervised learning methods to create a dense multidimensional space where words are represented by vectors. The position of such vectors is related to their semantic meaning, further developing the work on word embeddings (Bengio et al., 1994, 2003) which grounds on the idea of distributed representations for symbols (Hinton et al., 1986). The word embeddings are widely used in modern NLP since they allow for a dimensionality reduction compared to a traditional sparse vector space model. In Le and Mikolov (2014), expanding the previous work on word embeddings, it is presented a model capable of representing also sentences in a dense multidimensional space. Also in this case, sentences are represented by vectors whose position is related to the semantic content of the sentence. In such a space sentences with similar semantic meaning will be represented by vectors that are close to each other.

This recent rise of interest around text-based computational methods for measuring financial risk and distress is fueling a rapidly growing literature. The most covered area is sentiment analysis to be correlated with events of interest. Many of the previous approaches have been based on hand-crafted dictionaries that despite requiring work to be adapted to single tasks can guarantee good results due to the direct link to human emotions and the capability of generalizing well through different datasets. Examples of this kind are the papers of Soo (2013), Nyman et al. (2015), and Cerchiello et al. (2017, 2019, 2020). The first analyses sentiment trends in news narratives in terms of excitement/anxiety and find increased consensus to reflect pre-crisis market exuberance, while the second correlates the sentiment extracted from news with the housing market. In the last ones, particular source of information, Twitter data and Telegram chat, is employed within a graphical models framework and classification models, to highlight connectedness and potential systemic risk among bank and possible ICOs frauds. Despite the good results, there are applications where it could be preferable to avoid dictionaries in favor of more data driven methods, which have the advantage of higher data coverage and capability of going beyond single word sentiment expression. Malo et al. (2014) provide an example of a more sophisticated supervised corpus-based approach, in which they apply a framework modeling financial sentiment expressions by a custom data set of annotated phrases.

The study of bank defaults is important for two reasons. First, an understanding of the factors related to bank failures enables regulatory authorities to supervise banks more efficiently. If supervisors can detect problems early enough, regulatory actions can be taken, to prevent a bank from failing and reduce the costs of its bail-in or bail-out. Differently, those costs would be mainly faced by shareholders, bondholders and depositors in case of bail-in or governments and, ultimately, taxpayers in case of bail-out. Second, the failure of a bank can induce failures of other banks or of part of the financial system (Kaminsky et al., 2003; Scaramozzino et al., 2021). Understanding the determinants of a single bank failure may thus help to understand the determinants of financial systemic risks, were they due to microeconomic idiosyncratic factors or to macroeconomic imbalances. When problems are detected, their causes can be removed or isolated, to limit “contagion effects”. Most research papers on bank failures are based on financial market models, that originate from the seminal paper (Merton, 1974), in which the market value of bank assets is matched against bank liabilities. Due to its practical limitations, Merton's model has been evolved into a reduced form (see e.g., Vasicek, 1984), leading to a widespread diffusion of the resulting approach, and the related implementation in regulatory models. Indeed, the last few years have witnessed an increasing research literature on systemic risk rather than stress modeling, with the aim of identifying the most contagious institutions and their transmission channels. A comprehensive review is provided in Brunnermeier and Oehmke (2012).

We can state that, the employment of unstructured data in credit scoring analysis is a rather well-established approach. Cornee (2019) demonstrates the importance of textual information for exploiting the credit merit of 389 small and opaque businesses granted by a French social bank. Grunert et al. (2005) analyzing German SMEs, compare three different models: one based solely on financial data, another one on textual data and the last one on mixed data, demonstrating how the latter is the best in terms of predicting loan defaults. Such paper can be considered a direct and interesting comparison to our proposal, although in a different context. Djeundje et al. (2021) use psychometric data to estimate credit scoring models and show how these data can be used to predict good or bad client. They stress how helpful the approach can be, in particular in countries where financial information is not available. With the aim to improve access to credit for those people for whom it is not possible to obtain a credit history, Pedro et al. (2015) present a score, called Mobiscore, able to estimate the financial risk through mobile-phone data. From these data, it is possible to extract the applicant's personality and status which have proved to be capable of predicting the default like the classical data. Iyer et al. (2016) use hard information and soft information in peer-to-peer market. They show the importance of soft information in screening borrowers, in particular for borrowers with lower credit quality. Netzer et al. (2019) use the free text that borrowers write when applying for a loan to investigate the likelihood of default. They find that, from such text it is possible to detect intentions, emotional states and personality traits. They demonstrate how the integration of this type of data, with the financial ones, is able to improve the forecast of default up to 4.03%. In the context of peer-to-peer lending, Niu et al. (2019) study the impact of information obtained from social media on loan insolvency. Through three machine learning algorithms (random forest, AdaBoost, and LightGBM) they demonstrate that there is a significant correlation between such soft information and the default and that they can be used to improve default predictions. Finally, Guo et al. (2016) use a Latent User Behavior Dimension based Credit Model (LUBD-CM) for the applicant's credit analysis and show that this model significantly improves the forecast compared to standard models.

Our contribution aims at demonstrating the feasibility and usefulness of the integration of textual and numerical data into a machine learning framework for financial predictions. Thus, the goal of the predictive model is to correctly classify stressed banks from both financial news and financial numerical data. It differs from the previous described literature by considering financial institutions rather than borrowing clients. Thus, we account for a macro perspective instead of a micro data analysis. Indeed, we believe that exploiting methodologies which investigate the soundness and stability of the financial system as a whole is of paramount importance. Moreover, we investigate and evaluate the role played by deep learning techniques either as a tool for extracting information from textual data or as a proper mean for classification purposes (Pagnottoni, 2019).

The rest of the paper is organized as follows: in Section 2, we describe the machine learning framework with a discussion of the tuning parameters issue, in Section 3 we illustrate the data and the predictive task, in Section 4 we present the experimental results and in Section 5 we discuss the conclusions of the work with hints on future developments.

## 2. Methodology

Machine learning systems benefit from their ability to learn abstract representations of data, inferring feature representations directly from data instead of relying on manual feature engineering. This capability is particularly exploited in deep learning models, which provides flexibility and potentially better performance (Schmidhuber, 2015). These characteristics are crucial in Natural Language Processing tasks where the ability to generalize across languages, domains and tasks enhances the applicability and robustness of text analysis. The framework applied in this paper is an extension of the one developed in Rönnqvist and Sarlin (2017) with the aim of predicting banks' distress from textual data. Their approach infers banks distress conditions from textual news using a machine learning system based on two steps:

- The first step comprises an unsupervised algorithm to compute the semantic vectors associated to a specific news text. Dense vector representations of sentences mentioning target banks are learned using the Distributed Memory Model of Paragraph Vectors (PV-DM) by Le and Mikolov (2014) (here referred to as Doc2Vec). This algorithm represents each document by a dense vector which is trained to predict words appearing in the document. The semantic space obtained through the employed algorithm has a lower dimensionality (600 in this case) compared to a Bag of Words representation and encodes the word semantics. In such framework, in fact, words are represented by vectors whose distances reflect statistical properties of the language like synonymy, gender, verb tenses and many others. From this new space is easier to perform the classification task due to the reduced dimensionality and the wise positioning of the vectors that takes into account their semantic meaning.
- The second step of the framework performs a classification over the semantic vectors of sentences mentioning target banks through a supervised algorithm. The sentence representations are fed into a neural network classifier that is trained with distress event labels. The neural network architecture is constituted by an input layer with the same dimensionality of the semantic vectors (600 nodes), one hidden layer (50 nodes) and one output layer (2 nodes with stress prediction *e*∈{0, 1}) that returns the tranquil or distressed status prediction.

In this work we modify the previous model of Rönnqvist and Sarlin (2017) to integrate the financial numerical data and to evaluate the performance gain obtained by their combination with news data. The financial data that we integrate, contain information about bank accounting data, banking sector data and country macroeconomic data. In modifying the approach we kept the two-step structure of the previous framework. Thus, also in our case we previously compute the semantic representations of the textual data and then, in a second step, we classify the bank status. Anyway, between the two steps, we combine the semantic vectors with the numerical financial variables vectors. In this way, the classification performed in the second step takes into account both the information contained in the financial news and in the financial variables.

The approach used to learn the semantic vectors is a Distributed Memory Model Paragraph Vector (Le and Mikolov, 2014). In this model, the semantic vector representation is learned by training a feed forward neural network to predict the words contained in a document by their word context (previous *n* and following *n* words) and a randomly initialized semantic vector (sentence *ID*). The word contexts, used as features to predict the target words are fixed-length and sampled from a sliding window over the sentence. While training the network, the semantic vector gets updated by the training algorithm so that its representation positively contributes in predicting the next word and thus works as a semantic representation of the entire sentence (or text sequence). In this way, the sentence vector works as a memory for the model that once trained captures the semantics of continuous sequences. The sentence *ID*, in fact, can be thought of as an extra word representing the sentence as a global context on which the prediction of the next word is conditioned. Despite the random initialization of the semantic vectors, they gradually improve the capability of capturing the semantic of the sentence during the training. The training is performed by stochastic gradient descent with the gradients computed by the backpropagation algorithm. Formally, the training procedure seeks to maximize the average log probability:


(1) $$\begin{array}{l} \frac{1}{t+n}\overset{t-n}{\underset{i=1}{\sum }}\log\text{p}\left(w_{i+n+1}|s,w_{i},\ldots ,w_{i+n}\right) \end{array}$$

over the sequence of training words *w*_1_, *w*_2_, …, *w*_*t*_ in sentence *s* with word context of size *n*.

After being trained, the semantic vectors can be used as features for representing the sentence information content (e.g., in place of its Bag of Words representation). These features can be fed directly to conventional machine learning techniques such as logistic regression, support vector machines, neural networks or K-means clustering.

The algorithm can be used both to compute the semantic vectors of the sentences on which is trained and also to infer the semantic vectors of new unseen sentences. In the first case, the model learns the semantic vectors along with the word vectors from the training *via* backpropagation and gradient descent by minimizing the word prediction error on the training corpus. In the second case, the semantic vectors are calculated by gradient descent and backpropagation, while keeping the word vectors and the other model parameters fixed.

As first step, we compute the semantic representations of the sentences mentioning banks in our corpus. To obtain valid sentence representations for specific domains, it is important to train the model on large enough corpora that also contain task-specific texts. In our case we would like to capture both the general properties of the English language and the context specific terms and expressions related to banks. To this aim, we run the model on the entire corpus of ca. 262,000 articles that we have, disabling the sentence *ID* vector for those sentences that do not contain any bank occurrence. In this way the word representations, that the model internally builds, can take advantage of a larger quantity of text. The dimensionality of the semantic vectors (600) and the word context size of the algorithm (5) have been optimized by cross-validation.

**Figure 1:**
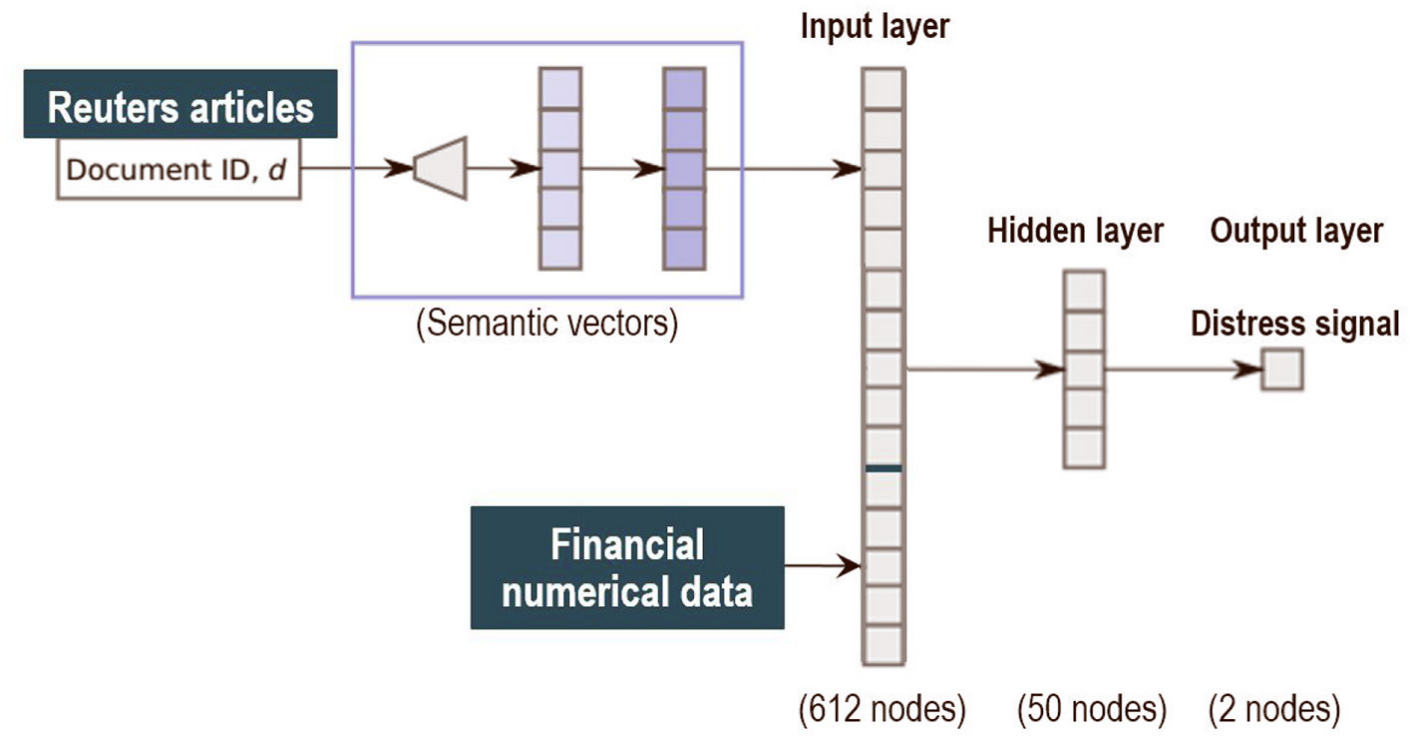
Structure of the model.

The second step performs the classification task on the combination of financial news and financial numerical information. It receives in input the news textual data on the banks, in the form of sentence level semantic vectors *V*_*s*_, and the vector of numerical financial data *F*_*s*_ for the corresponding bank in the same period. The classification model is a three layers fully connected feed forward neural network. The neural network has an input layer with 612 nodes, 600 input nodes for the semantic vector *V*_*s*_ and 12 input nodes for the numerical data *F*_*s*_. After the input layer, it has a 50 nodes hidden layer and a 2 nodes output layer with softmax activations *e*∈{0, 1} to encode the distress or tranquil status (see Figure 1). The network is trained by Nesterov's Accelerated Gradient Descent (Nesterov, 1983) to minimize the cross-entropy loss function. Hence, the objective is to maximize the average log probability:


(2) $$\begin{array}{l} \frac{1}{\left|S\right|}\underset{s\in S}{\sum }\log\text{p}\left(e_{s}|V_{s},F_{s}\right) \end{array}$$

In the trained network, the posterior probability *p*(*e*_*s*_ = 1|*V*_*s*_, *F*_*s*_) reflects the relevance of sentence *s* and the corresponding financial variables to the modeled event type.

### 2.1. Classifier Tuning

**Figure 2:**
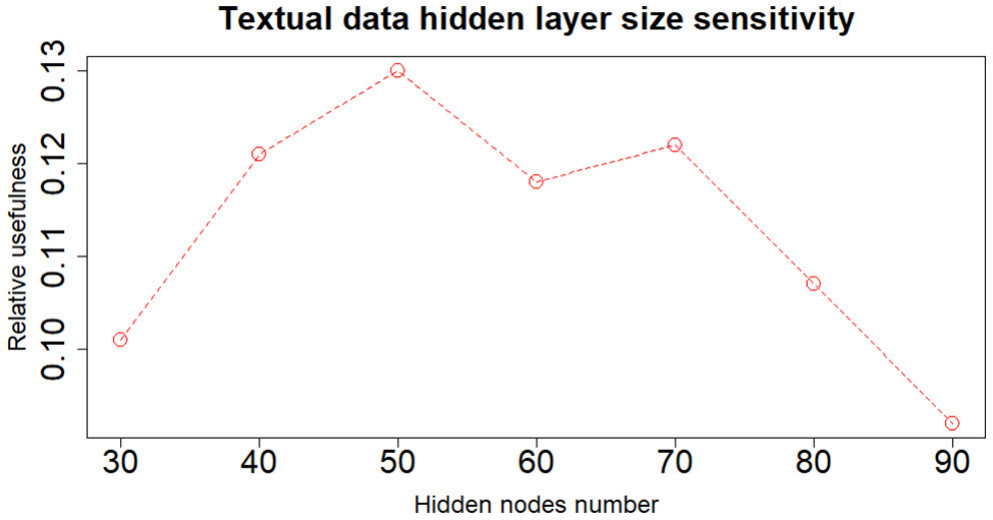
Textual data—sensitivity analysis on the number of nodes of the hidden layer.

**Figure 3:**
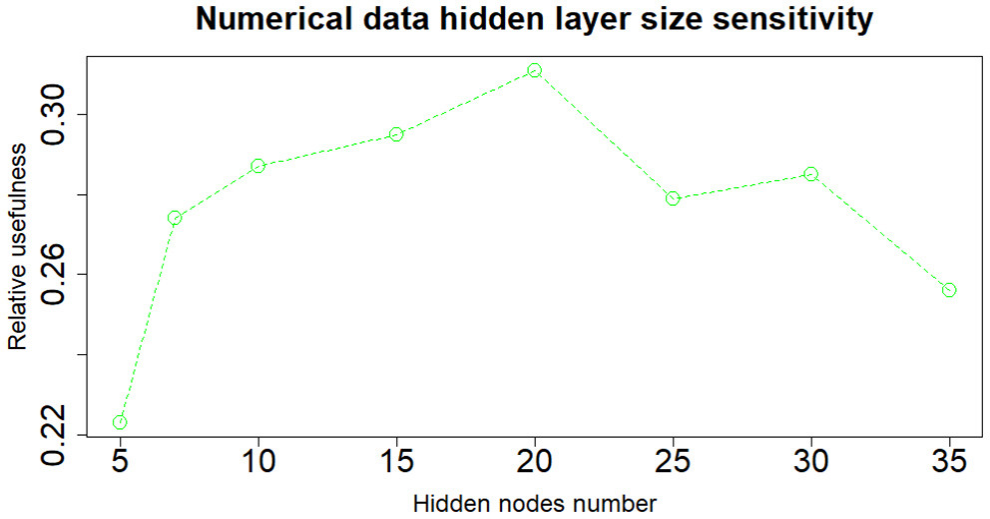
Numerical data—sensitivity analysis on the number of nodes of the hidden layer.

**Figure 4:**
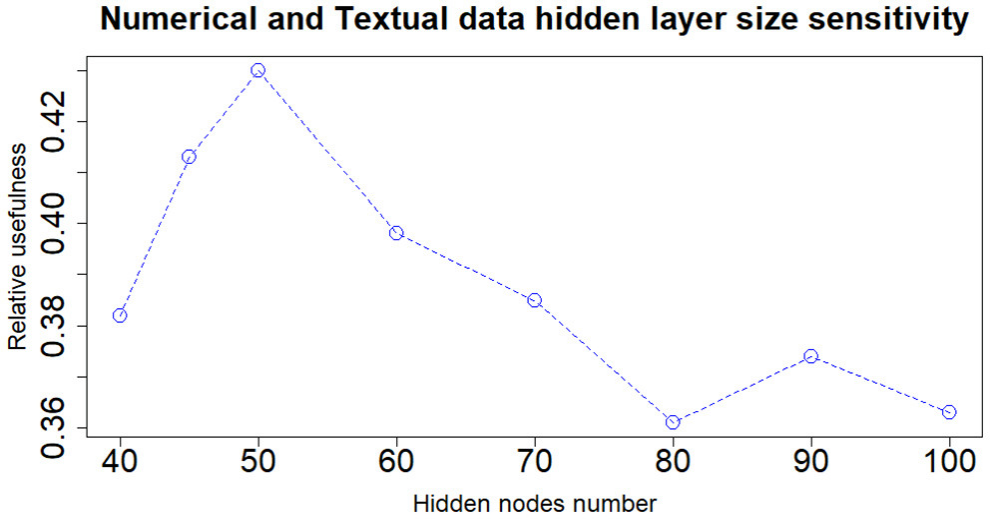
Numerical and textual data—sensitivity analysis on the number of nodes of the hidden layer.

When dealing with network model a key role is played by the architecture structure tuning. We have run a sensitivity analysis exploring different neural network configurations while training it with the Nesterov Accelerated Gradient Descent algorithm from Nesterov (1983). We have tested different hidden layers sizes, numbers of layers, learning rates, regularization parameters and dropout fractions (Hinton et al., 2012). For choosing the final network configuration, we applied the Occam's razor principle always preferring the simpler structure able to achieve a given performance. Thus, where performance is not reduced excessively, we try to select the network structure with fewer layers and fewer hidden nodes; this also helps to have better generalization and to reduce overfitting. In terms of hidden layers number, the network with one hidden layer (three layers in total including input and output) performs slightly better than those with more layers. We tested up to three hidden layers (5 layers in total, including input and output layers) and verified that the performance was monotonically decreasing. Regarding the number of hidden nodes, the network configuration that achieved the best relative Usefulness had 50 hidden nodes with a learning rate α of 5*e*-4 combined with an *L*_1_ regularization parameter λ of 1*e*-5. The parameter that mostly affects the results is the number of nodes in the hidden layer. Results of the sensitivity analysis on the hidden nodes number (with regards to one hidden layer network configuration) are reported in Figures 2–4, respectively for the case including textual data alone, financial numerical data alone and the combination of the two. The range of hidden nodes in the three sensitivities is different because the input vectors in the three cases have very different dimensions, 600 input nodes when considering only textual data, 12 input nodes when considering only numerical data and 612 input nodes when including both numerical and textual data. We do not investigate extensively the textual data case which has already been studied in Rönnqvist and Sarlin (2017). Regarding the numerical based case, we can notice that we have a range of hidden layer size comprised between 10 and 20 nodes where performances are stable and the relative Usefulness is around 30%. For the combined input (Numerical and Textual) we expected the right number of hidden nodes to be similar to the Textual data case since the input dimensionality is similar (600 and 612). In fact, we can see that there is a range around 50-60 hidden nodes where performance is stable around a relative Usefulness of 40%.

## 3. Data

As described in the previous section, we leverage two types of data. We rely on both textual and numerical data, aligned together by time and entities (banks), to classify bank distressed or tranquil conditions. The distress events dataset contains information on dates and names of the involved entities, related to the specific type of distress event to be modeled. The textual and financial numerical datasets accordingly contain respectively bank related articles and financial figures. The textual and numerical data are aligned and linked to a particular event matching the date and occurrences of the entity name within the sentence. For the financial numerical data with quarterly frequency, the date of the event is matched with the corresponding quarter. The model is then trained in a supervised framework to associate specific language and financial figures with the target bank status for the given period (tranquil or distressed).

### 3.1. Textual and Numerical Data

The textual data used in the study are part of a news articles database from Reuters on-line archive, ranging between 2007-Q1 and 2014-Q3. The original data set includes 6.6M articles, for a total of ca. 3.4B words. In order to select only articles related to the considered banks, we searched for banks names occurrences and selected only those articles with at least one occurrence. Banks names occurrences are identified using a set of patterns defined as regular expressions that cover common spelling variations and abbreviations of the banks names. The regular expressions have been iteratively developed on the data to increase accuracy, with a particular attention on avoiding false positives. As a result, from the entire corpus we retrieve ca. 262,000 articles mentioning any of the 101 target banks. Successively, the articles are split into sentences and only the sentences with the bank name occurrences are kept. We integrate financial contextual information with a database of distress related indicators for banks. The numerical dataset is composed of 12 variables for 101 banks over the period 2007–2014 with quarterly frequency. In Table 1, we list the considered numerical variables: bank-level balance sheets, income statement data, country-level banking sector data and macro-financial data.

**Table 1:** List of available numerical variables.

**Bank level**	**Bank sector level**	**Macro level**
Capital to asset	Mortgages to loans	House price gap (Deviation from trend of the real residential property price index)
Interest to liabilities	Securities to liabilities d4	Macroeconomic Imbalance Procedure (MIP), international investment position
Reserves to asset	Financial assets to gdp	Private debt
-	-	Government bond yield
-	-	Credit to gdp
-	-	Credit to gdp delta over 12 months

The three bank-specific variables are: the ratio of tangible equity to total assets, the ratio of interest expenses to total liabilities and the NPL reserves to total assets ratio. The three banking-sector features are: the mortgages to loans ratio (4-months change), the ratio of issued debt securities to total liabilities (4-months change) and the ratio of financial assets to GDP. The six Macro financial level features are: the House price gap (Deviation from trend of the real residential property price index filtered with the Hodrick-Prescott Filter Hodrick and Prescott, 1980 with a smoothing parameter λ of 1,600), the international investment position from the ECB Macroeconomic Imbalance Procedure (MIP) Scoreboard, the country private debt, the government bond yield (4-months change), the credit to GDP ratio and the credit to GDP 1-yr change.

In Table 2, we report summary statistics of the considered numerical variables.

**Table 2:** Summary statistics of available numerical variables.

**Variable**	**Mean**	**Variance**	**Standard Deviation**	**Kurtosis**
Capital to asset	2.5	10.2	3.2	21.5
Reserves to asset	4.2	8.5	2.9	4.3
Interest to liab	3.4	8.5	2.9	104.6
Financial assets to gdp	385.0	134365.2	366.6	33.2
Mortgages to loans d4	0.2	1.7	1.3	0.1
Securities to liab d4	−12.0	1342234.9	1158.5	105.7
Credit to gdp	140.2	2623.4	51.2	0.0
Credit to gdp d12	13.7	479.1	21.9	0.5
House price index rt16 gap	−2.5	33.7	5.8	6.8
International investment position	−21.0	2967.7	54.5	0.0
Private debt	188.2	4938.7	70.3	0.1
Gov bold yield d4	0.0	11.4	3.4	23.5

Then, we match the distress events with the available textual news data. The events comprehends bankruptcies, direct defaults, government aid and distressed mergers as presented in Betz et al. (2014). The distress events in this dataset are of three types. The first type of events include bankruptcies, liquidations and defaults, with the aim of capturing direct bank failures. The second type of events comprises the use of state support to identify banks in distress. The third type of events consists of forced mergers, which capture private sector solutions to bank distress. The inclusion of state interventions and forced mergers is important to better represent bank distress since there have been few European direct bank failures in the considered period. Bankruptcies occur if a bank net worth falls below the country-specific guidelines, whereas liquidations occur if a bank is sold and the shareholders do not receive full payment for their ownership. Defaults occur if a bank failed to pay interest or principal on at least one financial obligation beyond any grace period specified by the terms or if a bank completes a distressed exchange. The distress events are formally considered to start when a failure is announced and to finish at the time of the “de facto” failure.

A capital injection by the state or participation in asset relief programs (i.e., asset protection or asset guarantees) is an indication of bank distress. From this 'indicator' are excluded liquidity support and guarantees on banks' liabilities, since they are not used for defining distressed banks. The starting dates of the events refer to the announcement of the state aid and the end date to the execution of the state support program. Distressed mergers are defined to occur if (i) a parent receives state aid within 12 months after a merger or (ii) if a merged entity exhibits a negative coverage ratio within 12 months before the merger. The dates for these two types of distress events are defined as follows, respectively: (i) the starting date is when the merger occurs and the end date when the parent bank receives state aid, and (ii) the start date is when the coverage ratio falls below 0 (within 12 months before the merger) and the end date when the merger occurs. Thus far, data at hand assign a unique label for the stress events, not allowing a more detailed descriptive summary of the three event types.

### 3.2. Data Integration

The following step in the data preparation has been the addition of numerical financial data to the text news database. The numerical data were aligned with the set of sentences in which bank names occurred. The purpose was to match each and every mention of a bank with the corresponding numerical financial data aligned according to the same time horizon. Since the news and the financial data have different frequency, in particular, news have higher frequency while financial data are reported quarterly, the latter are replicated several times to match with the former. For each news regarding a bank within a given quarter, financial data are replicated and appended to the semantic vector of the news. The alignment between numerical and textual data resulted in the removal of some banks from the dataset due to missing data, causing a reduction from 101 to 62 target institutes (Table 3) and from about 601,000 to 380,000 news sentences.

**Table 3:** List of considered financial institutions.

**Financial institution**	**Country**	**Financial institution**	**Country**	**Financial institution**	**Country**
Aareal Bank	DE	Carnegie Investment Bank	SE	Kommunalkredit	AT
ABN Amro	NL	Commerzbank	DE	LBBW	DE
Agricultural Bank of Greece	GR	Credit Mutuel	FR	Lloyds TSB	UK
Allied Irish Banks	IE	Credito Valtellinese	IT	Max Bank	DK
Alpha Bank	GR	Cyprus Popular	CY	Monte dei Paschi di Siena	IT
Amagerbanken	DK	Danske Bank	DK	National Bank of Greece	GR
ATE Bank	GR	Dexia	FR	Nordea	SE
Attica Bank	GR	EBH	DK	NordLB	DE
Banca Popolare di Milano	IT	EFG Eurobank	GR	Nova ljubljanska banka Group (NLB)	SI
Banco Popolare	IT	Erste Bank	HU	OTP Bank Nyrt	HU
Bank of Cyprus Public Co Ltd	CY	Fionia (Nova Bank)	DK	Piraeus Bank	GR, CY
Bank of Ireland	IE	Fortis Bank	LU, NL, BE	Pronton Bank	GR
Banque Populaire	FR	HBOS	UK	RBS	UK
Bawag	AT	Hellenic	GR	Roskilde Bank	DK
BayernLB	DE	HSH Nordbank	DE	Societe Generale	FR
BBK	ES	Hypo Real Estate	DE	Swedbank	SE
BNP Paribas	FR	Hypo Tirol Bank	AT	T-Bank	GR
BPCE	FR	IKB	DE	UNNIM	ES
Caixa General de Depositos	PT	ING	NL	Vestjysk	DK
Caja Castilla-La Mancha	ES	Irish Nationwide Building Society	IE		
CAM	ES	KBC	BE		

After cleaning the dataset, numerical data have been normalized with a standard approach by subtracting the mean value from each numerical variable of the dataset and dividing it by the standard deviation. The resulting input vector for the 612 dimensional input layer of the neural classifier receives in input a 612 dimensional vector obtained from the concatenation of the 600-dimensional semantic vector coming from the unsupervised modeling, described in the Section 2, with the 12-dimensional numerical financial data vector. The dataset is then split into five folds, three for training, one for validation and one for testing according to a cross-validation scheme. The folds are created so that all the data regarding a given bank are in the same fold.

The framework we apply is composed of an unsupervised algorithm and a supervised neural network classifier. To train the classifier, a label indicating the distressed or tranquil status of the bank is provided. The dataset has been labeled according to the bank status with 0 indicating tranquil and 1 distressed. The proportions of the two classes are highly unbalanced: 93% of the data-points is associated to a tranquil status and only the remaining 7% is associated to distress events. Such imbalance of the classes has a significant impact both on the training and on the evaluation of the model. Regarding the training, it is important that the model is able to generalize also from the few distress examples, while for the evaluation it could be useful to include other performance measures and not just the accuracy. Indeed, a trivial model that always predicts the tranquil status would achieve a 93% accuracy, thus it would be interesting to measure the improvements against this baseline. Moreover, the user is likely interested in weighting differently first error and second error types, especially if we consider potential early warning applications of this model. The usefulness measure, introduced in Sarlin (2013), satisfies such requirements.

## 4. Results

The experimental results confirm that the integration of numerical and textual data amplifies the prediction capability of the model compared to the inclusion of only textual data. The distress events in the database represent only 7% of the cases, resulting in very skewed training classes as explained earlier. Moreover, given the nature of the problem, the identification of distress situations, it could be useful to weight differently false positives and false negatives. In an early warning application, a sensitive system is often preferable since a further investigation phase follows the detection of a warning. These peculiarities have to be taken into account during the evaluation of the model.

### 4.1. Evaluation and Experimental Results

For the evaluation of our model, we resort to the relative usefulness as measure of performance. The relative usefulness (*U*_*r*_), introduced in Sarlin (2013) is a measure that allows to set the error type preference (μ) and to measure the relative performance gain of the model over the baseline compared to the performance gain over the baseline of a perfect model. The index is computed starting from the probabilities of the true positive (*TP*), false positive (*FP*), true negative (*TN*) and false negative (*FN*). By using these quantities, we can define the model loss *L*_*m*_ (Equation 4) and a baseline loss *L*_*b*_ set to be the best guess according to prior probabilities *p*(*obs*) and error preferences μ (Equation 3).


(3) $$L_{b}=min\{\begin{array}{l} \mu *p(obs=1)\\ \left(1-\mu )*p(obs=0\right) \end{array}$$

(4) $$\begin{array}{l} L_{m}=\mu *p\left(FN\right)+\left(1-\mu \right)*p\left(FP\right) \end{array}$$

The absolute Usefulness (*U*_*a*_) and the relative Usefulness (*U*_*r*_) are directly derived from the loss functions:


(5) $$\begin{array}{l} U_{r}=\frac{U_{a}}{L_{b}}=\frac{L_{b}-L_{m}}{L_{b}} \end{array}$$

The absolute Usefulness *U*_*a*_ of a model corresponds to the loss “generated” by the model subtracted from the loss of ignoring it *L*_*b*_. From Equation 5 we can see that the relative usefulness is equal to 1 when the model loss (*L*_*m*_) is equal to 0, thus when the model is a perfect classifier. As a consequence, the relative usefulness measures the gain over the baseline compared to the gain that an ideal model would achieve. *U*_*r*_ reports *U*_*a*_ as a percentage of the Usefulness that one would gain with a perfectly performing model. This measure highlights the fact that achieving well-performing, useful models on highly imbalanced data is a difficult task. To compute the relative usefulness (*U*_*r*_), we have set the error type preference (μ) equal to 0.9 in accordance with the indications of previous studies like Betz et al. (2014) and Constantin et al. (2016) on the importance of signaling every possible crisis at cost of some false positive (*FP*) (setting μ = 0.9 we are implying that missing a crisis is about 9 times worse than falsely signaling one). This is especially true if following the warning signal, a further investigation action is triggered. To evaluate distress condition of a bank over a period, the predictions are aggregated on a monthly basis by bank entity. This is done by averaging the predictions at the single sentence level by month for each different bank. This has been done to take into account the textual information available over the past month period. As a result of this procedure, the classification task can be summarized as understanding which banks are in distress status month by month, based on the news sentences and numerical data available over the previous month.

**Figure 5:**
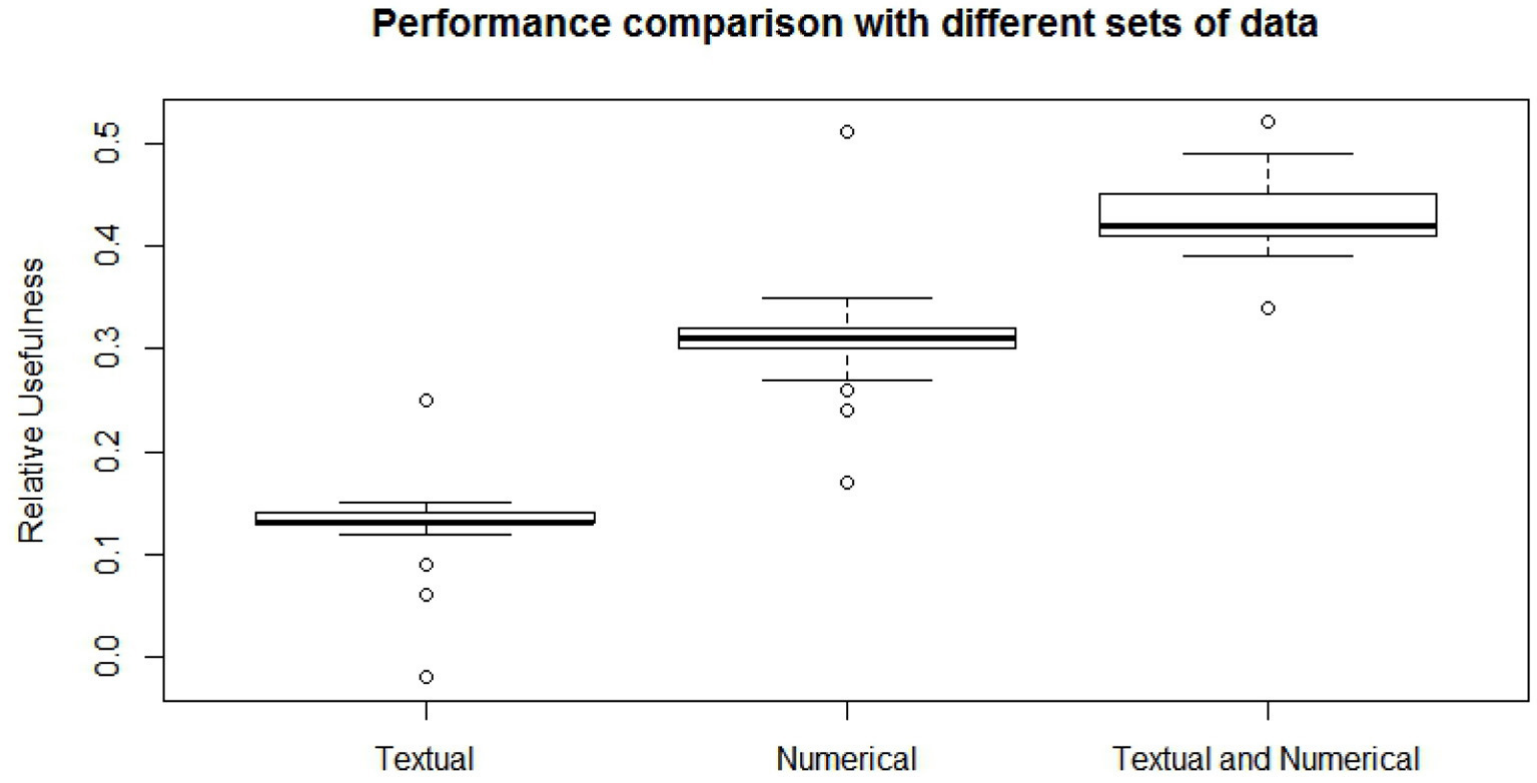
Comparison of the relative usefulness obtained with the textual financial data **(left)**, numerical financial dataset **(center)**, and with their combination **(right)**.

To evaluate the model on this classification task, we have trained it fifty times on the same dataset, recording the relative usefulness (*U*_*r*_) result after each run and then averaging them. For each of the fifty trainings, the folds are resampled and the neural net is randomly initialized. To quantify the gain obtained from merging numerical and textual data we have done three different experiments, training the model respectively with textual data only (Figure 5, left), numerical data only (Figure 5, center) and numerical and textual data together (Figure 5, right). As it appears from Figure 5, the case with textual data alone achieves an average relative usefulness of 13.0%, while the case with numerical data alone shows an average relative usefulness of 31.1%. The combination of these two dataset and their exploitation in the model grants an average relative usefulness of 43.2%, thus it positively enhances the prediction capability of the model. From these results we can also understand that, as expected, the financial numerical data hold the majority of the informative potential necessary for the labeling task but that the addition of textual information provides a non-negligible 12.1% improvement to the relative usefulness of the model.

## 5. Conclusions

In this paper, we have presented an approach for the integration of financial numerical data and financial news data into a single machine learning framework. The aim is to improve performances of bank distress conditions identification through the combination of these two data sources. The implemented model processes textual data through an unsupervised neural network model, Doc2Vec, converting the documents sentences into sentence vectors. The derived sentence vectors are then concatenated with the financial numerical data to form a single input vector. Each of these vectors becomes the input to a supervised classifier, a three layers fully connected neural network.

The classification task was characterized by a high data imbalance among the classes which poses concerns for both the model training and evaluation. The implemented model has shown to be able to learn the combinations of banks' financial conditions and news semantic content that are more frequently associated with distress conditions. This is reflected in the improved performance obtained when including both news data and financial numerical data as input to the model. In this case, indeed, the model achieves an average relative usefulness of 43.2%, compared to 31.1% when using only numerical data and 13.0% when utilizing only textual data. The sensitivity analysis performed on the model supports these results indicating stability within a certain range of architectures.

### 5.1. Future Works

Some limitations of this model reside in the way the news are processed and converted into vectors and how they are fed to the network to classify the distress. Methods like Doc2Vec with Distributed Memory approach in fact, while not using a pure bag of word approach, still ignore important text information to truly understand a sentence and not only its topic or its average sentiment. For example, long range dependencies in the text are not considered and polysemous words and mixed word polarities can also affect the performance of this algorithm. Moreover, Doc2Vec performs significantly better when trained on a large quantity of text similar to the application domain. This quantity of text was available in our study but could pose a limit to applications in niche specific domains or its extension to less widespread languages. In the last years, there have been many improvements in the NLP field that can help to overcome these limitations. A particularly interesting class of models is the so-called Sequence to Sequence RNNs, that recently have become very popular. These models are composed of two RNNs (one encoder and a decoder) that are trained in an unsupervised setting to reconstruct their own input text. Sequence to Sequence architectures pre-trained on financial and bank related text could be used as a substitute for the Doc2Vec representation in our approach. Differently from Doc2Vec these models consider explicitly the word order and long range dependencies over the entire text input sequence. As a result they can provide more accurate text vector representations. Furthermore, it is possible to augment the model capability providing few additional manually engineered features like a gazette of words with positive/negative polarity from a financial stability point of view.

An additional future work direction that could improve this framework as an early warning tool would be considering the news dynamic evolution (Cerchiello et al., 2018). In the present paper, news are aggregated at the monthly level, thus sub-monthly dynamics are lost. Using a RNN as distress classifier, it would be possible to sequentially feed the news vectors into the network. In this way, considering daily or weekly news aggregation, it would be possible to take into account also these dynamic effects (e.g., overall negative sentiment but with a positive trend in the last weeks).

The methodology here applied is general and extensible to other problems were the integration of text and numerical covariates can improve classification and early warning performances. Similarly interesting results are to be expected in areas where textual data hold information with higher granularity and frequency, directly influencing the data to be predicted in the short run like in the case of financial markets.

## Data Availability Statement

The original contributions presented in the study are included in the article/supplementary materials, further inquiries can be directed to the corresponding author/s.

## Author Contributions

GN prepared the data, run the analysis, and wrote Sections 2 and 4. PS provided the data and wrote the relative description. SR wrote the preliminary version of the code. PC wrote the Sections 1 and 5 and supervised the overall process. All authors contributed to the article and approved the submitted version.

## Conflict of Interest

The authors declare that the research was conducted in the absence of any commercial or financial relationships that could be construed as a potential conflict of interest.

## Publisher's Note

All claims expressed in this article are solely those of the authors and do not necessarily represent those of their affiliated organizations, or those of the publisher, the editors and the reviewers. Any product that may be evaluated in this article, or claim that may be made by its manufacturer, is not guaranteed or endorsed by the publisher.
